# External Marginal Gap Variation and Residual Fracture Resistance of Composite and Lithium-Silicate CAD/CAM Overlays after Cyclic Fatigue over Endodontically-Treated Molars

**DOI:** 10.3390/polym13173002

**Published:** 2021-09-04

**Authors:** Andrea Baldi, Allegra Comba, Riccardo Michelotto Tempesta, Massimo Carossa, Gabriel Kalil Rocha Pereira, Luiz Felipe Valandro, Gaetano Paolone, Alessandro Vichi, Cecilia Goracci, Nicola Scotti

**Affiliations:** 1 Department of Surgical Sciences, Dental School Lingotto, University of Turin, 10126 Turin, Italy; Andrea.baldi@unito.it (A.B.); alle_comba@yahoo.it (A.C.); riccardo.tempesta@hotmail.it (R.M.T.); carossamassimo@gmail.com (M.C.); 2 Department of Restorative Dentistry, Division of Prosthodontics, Federal University of Santa Maria, Santa Maria 97105-900, Brazil; gabrielkrpereira@hotmail.com (G.K.R.P.); lfvalandro@gmail.com (L.F.V.); 3 Department of Dentistry, IRCCS San Raffaele Hospital and Dental School, Vita Salute University, 20158 Milan, Italy; gaetano.paolone@gmail.com; 4 Dental Academy, University of Portsmouth, William Beatty Building, Hampshire Terrace, Portsmouth PO1 2QG, UK; alessandrovichi1@gmail.com; 5 Department of Medical Biotechnologies, University of Siena, 53100 Siena, Italy; cecilia.goracci@unisi.it

**Keywords:** micro-CT, 3D gap, fracture resistance, fiber post, overlay

## Abstract

The purpose of this in vitro study was to evaluate the external marginal gap variation with a 3D quantitative method and the residual fracture resistance after cyclic fatigue in endodontically treated molars restored with overlays of different materials, with and without fiber posts-supported buildups. Forty-eight human maxillary molars were selected, endodontically treated, prepared with standardized MOD cavities and randomly allocated into 6 study groups considering the “core strategy” (build-up with composite resin; build-up with composite resin supported by a fiber post); and the “restorative material” of the indirect adhesive overlay (GrandioBlocks, Voco; Cerasmart, GC; CeltraDuo, Dentsply). All procedures were executed according with manufacturers guidelines. Micro-CT analysis prior and after cyclic fatigue were executed, followed by scanning electron microscope analysis and fracture resistance test. The Two-Way ANOVA analysis showed that interfacial gap progression was significantly influenced by the “core strategy” (*p <* 0.01) but not of “restorative material” (*p =* 0.59). Concerning fracture resistance, “restorative material” was statistically significant (*p <* 0.01), while “core strategy” (*p =* 0.63) and the interaction (*p* = 0.84) were not. In conclusion, the fiber post presence within the build-up promoted a lower interfacial gap opening after fatigue, evaluated through micro-CT scans. In terms of fracture resistance, teeth restored with Cerasmart and Celtra Duo were statistically similar, but superior to GrandioBlocks.

## 1. Introduction

Preservation of healthy dental tissue is a fundamental factor in the longevity of restorations, especially when dealing with endodontically treated teeth (ETT), whose mechanical failure by fracture is more common compared to vital ones [1,2]. This increased fragility is strictly related to the pathology itself, but also to the procedures performed to devitalize and restore the tooth. On the other hand, pulp vitality loss, effects of irritants, medicaments and bacteria seem to play a secondary role on the fracture resistance [3,4]. In the past, there was the opinion that ETT needed a root canal post and full coverage crown rehabilitation [5]. Aquilino and Caplan showed that cuspal coverage could increase up to six times the survival rate of non-vital posterior teeth [6]. Therefore, the full crown has been considered the gold standard therapeutic approach for large cavities in ETT for years [7]. However, full crown preparations tend to remove a large amount of healthy dental tissue from teeth that have already lost a huge quantity of sound tooth structure due to pathology and endodontic procedures [8]. Hence, the majority of recent studies have focused more on partial direct or indirect bonded restorations, which ensure higher sound tissue preservation than traditional fixed full crowns [9]. It has recently been demonstrated that onlays, overlays and endocrowns can equally be effective compared to traditional crowns, in terms of mechanical, functional and esthetics properties, while simultaneously preserving tooth structure [10,11]. For these restorations, the use of different materials has been successfully proposed, such as glass-reinforced ceramics, resin composites, and hybrid ceramics [12,13,14]. These materials, which can be all processed through CAD/CAM workflows, showed good performance in both in vitro and in vivo studies [14,15,16].

To improve mechanical properties of the tooth-restoration complex, fiber posts have been indicated in association with direct restorations as well as during buildup procedures which support indirect adhesive restorations [17,18]. A recent study by Ausiello et al. showed, through a FEA analysis, how a hybrid composite post should be sufficient to optimize the stress distribution, dissipating stress from the coronal to the apical end [19]. Kemaloglu et al. (2015) showed that a fiber network might change stress dynamics at the interfaces [20] and recent studies suggested that this fact might influence marginal gap progression [21,22]. Different results concerning their effect on fracture resistance have been reported in literature [23,24,25], and it has been suggested that fiber reinforced materials might lower the number of clinically unrepairable fractures [26] even if ferrule effect must be considered as primary importance [19,27].

Literature clearly supports that ETT, especially posterior ones, should be treated with cuspal coverage restorations to increase fracture resistance [28]. It has also been reported that adhesive indirect restorations are able to well-transmit and distribute functional stresses to dental hard tissues, potentially reinforcing the weakened tooth structure while preserving sound tissues [25,29]. However, despite the significant development of adhesive protocols [30] and restorative materials, failures related to secondary caries, restoration fracture or debonding are still a major issue when dealing with indirect partial adhesive restorations on ETT [16,31]. It should be considered that, upstream of a catastrophic failure, the marginal gap formation could potentially lead to secondary caries formation, and also contribute for lowering tooth resistance [11,32,33]. Leakage can be caused by several factors: the volume reduction of the luting cement related to chain assembling generates tensile forces and subsequent stress-relieving gaps which could appear inside the tooth-restoration interface. If these gaps exceed a width of approximately 60 μm at the outer margin of the restoration, an increment of postoperative sensitivity and secondary caries might be reported [34]. Furthermore, during oral function, the tooth-restoration complex is exposed to fatigue stress derived from cyclical intermittent loading with the progressive onset of marginal leakage [35]. Consequently, the analysis of marginal degradation is today crucial to better understand biomechanical failures that could occur clinically.

Despite the presence in literature of a great number of in vitro studies which focuses on resistance of direct and indirect adhesive solutions on ETT [36], there are few papers regarding the tooth-restoration interface behavior of bonded cuspal coverage rehabilitations after exposition to cyclic intermittent loading. Considering the importance to study the effect of fatigue on the external margins of an adhesive restoration, the aim of the present in vitro study was to evaluate the tridimensional marginal gap and the consequent fracture resistance after cyclic fatigue in ETT restored with overlays of different CAD/CAM materials, with and without fiber posts-supported buildups (FPSbu). The initial null hypotheses are that both marginal gaps opening, and fracture resistance are not influenced (1) by the presence/absence of a FPSbu and (2) by the CAD/CAM material employed.

## 2. Materials and Methods

### 2.1. Study Design

The general description of the main materials used in the present study, their manufacturers and composition are listed in Table 1.

This study was designed in 6 study groups (n= 8), where the specimens were randomly allocated (www.randomizer.org) considering:

(i). “Core build-up” in 2 levels, being one condition where the build-up core was done only using a bulk-fill composite resin (Grandioso X-tra, Voco, Cuxhaven, Germany); or another condition where it was done associating composite resin and a fiber post (Rebilda Post #15, Voco, Cuxhaven, Germany);
(ii). “CAD/CAM blocks” in 3 levels: after core build-up, 3 different CAD/CAM restorative materials were tested: a nanohybrid composite resin (GB, GrandioBlocks, Voco, Cuxhaven, Germany), a flexible hybrid ceramic (CS, Cerasmart 270, GC, Tokyo, Japan), or a zirconia reinforced lithium silicate (CD, Celtra Duo, Dentsply, Konstanz, Germany).

### 2.2. Specimen Preparation

Forty-eight (n = 48) human upper maxillary molars with mature apices, extracted for periodontal reasons within the last 4 months, were selected and stored in distilled water at room temperature. The inclusion criteria were as follow: sound teeth, similar root (length > 12 mm) and crown size (10 mm ± 2 mesio-distal, 10 mm ± 2 bucco-oral) and no crack or demineralization under visual examination with light trans-illumination and magnification. Ultrasonic scaling and polishing were performed for surface debridement. All samples were collected with informed consent in the Department of Cariology and Oper Dent, University of Turin. The ethical committee of the University of Turin approved the study protocol (DS_00071_2018).

Endodontic treatment was carried out in all specimens by the same expert operator (Pathfiles 1-2-3 and ProTaper Next X1-X, Dentsply Maillefer, Ballaigues, Switzerland) to the working length, set at 1 mm short of the visible apical foramen. Irrigation was performed with 5% NaOCl (Niclor 5; Ogna, Muggiò, Italy) alternated with 10% EDTA (Tubuliclean, Ogna, Milan, Italy). Thereafter, specimens were obturated with gutta-percha points (GuttaPercha Points Medium, Inline; B.M. DentaleSas, Turin, Italy) using down Pack (Hu-Friedy, Chicago, IL, USA) and an endodontic sealer (Pulp Canal Sealer EWT; Kerr, Orange, CA, USA). After that, gutta-percha backfilling was performed (Obtura III system, Analytic Technologies, Redmond, WA, USA).

A single and trained operator prepared the standardized MOD cavities setting residual wall thickness of buccal and oral cusps at the height of the contour to 1.5 ± 0.2 mm and placing mesial and distal cervical margins 1 mm coronally to the CEJ. For cavity preparation, cylindrical diamond burs (model 835KR; Komet, Schaumburg, IL, USA) under copious air-water cooling were used in a high-speed headpiece (Kavo Dental GmbH, Biberach, Germany). All internal edges were then smoothed and rounded with an Arkansas point (FG 645, Komet, Schaumburg, IL, USA), in order to remove non-sustained enamel.

Considering the factor “core build-up”, samples were divided in 2 groups (n = 24) according to the build-up technique. In the first group (G1) cavities were subjected to the following adhesive procedure: Selective enamel etching 30 s with 35% phosphoric acid (K-etchant, Kuraray Noritake Dental, Tokyo, Japan), rinsing 30 s and air-drying. A universal adhesive system (Futurabond U, Voco, Cuxhaven, Germany) was applied in self-etch mode following the manufacturer instruction and light-cured for 20 s with a LED light curing unit at 1000 mW/cm^2^ (Cefalux 2, Voco, Cuxhaven, Germany). The MOD cavity was horizontally incrementally restored with a bulk fill material (Grandioso X-Tra, Voco, Cuxhaven, Germany). Each layer, maximum 3 mm thick, was light cured with the same curing LED lamp for 30 s.

In the second group (G2) a single 8 mm post-space was prepared in the palatal root employing dedicated drills (Rebilda Post #15 Voco, Cuxhaven, Germany). The correct length and adaptation of each post (Rebilda Post #15 Voco, Cuxhaven, Germany) was verified. Post spaces were then rinsed and dried with paper points, while fiber posts were cleaned with ethanol for 30 s. A universal adhesive system (Futurabond U, Voco, Cuxhaven, Germany) was applied on post spaces and over each fiber post following the manufacturer instruction and light-cured for 20 s. Dual-cure luting cement (Bifix QM, Voco, Cuxhaven, Germany) was applied according to the manufacturer’s instructions and injected into the post-space with a suitable sized mixing tip. Fiber posts were slowly inserted into the post-space and the excess cement was removed. Each specimen was light cured for 2 min using the same LED lamp and a composite build-up was performed as described for G1.

In each sample, 360° enamel margins were exposed with an overlay beveled preparation, in order to obtain 2 mm space for the restoration. In order to standardize preparations, an initial anatomical occlusal reduction was performed with 1.8 mm diameter cylindric bur (model 835KR; Komet, Schaumburg, IL, USA). After that, mesial and distal boxes were prepared with dedicated sonic points (n°34 and n°35, SonicFlex, Kawo, Shangai, China), cervically exposing enamel and remaining 1 mm above CEJ level. Occlusal sharp edges were beveled with a football-shaped bur angulated at 45° (model 8379-021, Komet, Schaumburg, IL, USA). Finishing was performed with same-shape burs with fine and extra-fine grit, then Arkansas (FG 645, Komet, Schaumburg, IL, USA) and rubber points were used to smooth all the corners. Specimens were then scanned with an intraoral camera (Cerec Omnicam AC, Dentisply, Sirona, Konstanz, Germany) and each group was divided in 3 subgroups (n = 8) according to the CAD/CAM restorative material: a nanohybrid composite (Grandio Blocks, Voco, Cuxhaven, Germany; GB), a flexible hybrid ceramic (Cerasmart 270, GC, Tokyo, Japan; CS), and a zirconia reinforced lithium silicate (Celtra Duo, Dentsply, Konstanz, Germany; CD). All overlays were designed with a CAD system, that allowed to standardize a 2 mm thickness of the restorations (Cerec 4.5.2 software, Dentisply, Sirona, Konstanz, Germany) and milled with material-specific default settings in extra-fine mode (Cerec MC XL, Dentsply, Sirona, Konstanz, Germany). Once milled, zirconia reinforced lithium silicate was crystallized (Cerec Speedfire, Dentisply, Sirona, Konstanz, Germany) according to the manufacturer instructions. Each overlay was then luted with universal adhesive (Futurabond U, Voco, Cuxhaven, Germany) and a dual-curing cement (Bifix QM, Voco, Cuxhaven, Germany) following manufacturer instructions. After overlay adaptation and cement excess removal with brushes, light curing was performed for 60 s for each side with the same LED lamp. A final 20 s/side polymerization was performed after covering the specimen with transparent air barrier gel. Finishing and polishing with diamond burs and silicone cups was performed to obtain a perfectly smooth surface.

**Figure 1:**
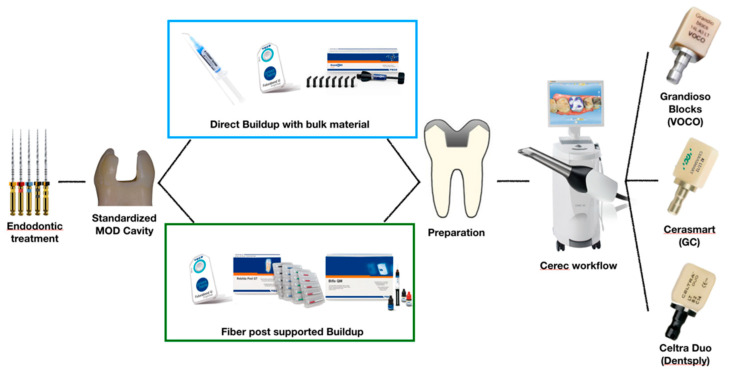
Specimen preparation workflow.

A summary of the specimen preparation protocol is presented in Figure 1.

### 2.3. Micro-CT Scanning

Specimens were scanned with X-ray micro computed tomography (micro-CT) for high-resolution scans (SkyScan 1172 Micro-CT, Bruker, Billerica, MA, USA), using following parameters: Voltage = 100 kV, current = 80 A, source-object distance = 80 mm, source-detector distance = 220 mm, pixel binning = 292, exposure time/projection = 3; aluminum and copper (Al + Cu) filter; pixel size = 10 µm; averaging = 5; rotation step = 0.4°. Images were reconstructed (NRecon, Bruker, Billerica, MA, USA) to obtain DICOM files, with standardized parameters: beam hardening correction = 25%, smoothing = 3, ring artifact reduction = 7. The same procedure, with the same parameters, was performed after cyclical intermittent loading in order to maintain consistency between data.

### 2.4. Chewing Simulation

Specimens were subjected to cyclic intermittent loading in distilled water using a CS-4.4 chewing simulator (SD Mechatronik, Feldkirchen-Westerham, Germany). A 50 N force was applied using 6 mm diameter steatite balls as antagonist, accordingly to previous studies on fatigue testing [25,26], with the following settings: frequency = 1 Hz, speed = 16 mm/s, sliding = 2 mm over the buccal triangular crest, number of cycles = 500,000.

### 2.5. Scanning Electron Microscope Analysis

Twelve specimens, two for each subgroup, were randomly selected after mechanical aging and cleaned in an ultrasonic bath with alcohol (TUC-150; Telsonic AG, Bronschhofen, Switzerland) for three minutes and then air-dried. Polyvinylsiloxane impressions were taken (Flexitime Light Flow, Heraeus Kulzer) and poured with epoxy resin (EpoFix; Struers) to produce replicas, which were mounted on aluminum stubs and sputter-coated (100 s, 50 mA) with gold/palladium by use of a sputter coating device (Balzers SCD 050; Balzers, Liechtenstein). Replicas were examined under a scanning electron microscope (Emission Scanning Electron Microscopy, Zeiss Supra 40 Field). Different magnification (66×; 150×; 500×; 1000×) images were obtained with following settings: WD = 10 mm, aperture size = 30.00 μm, EHT = 5.00 kV, signal A = In Lens, stage at T = 0°.

### 2.6. Fracture Resistance Test

Specimens were submitted to static fracture resistance test using a universal testing machine (Instron, Canton, MA, USA) with a 6 mm diameter steel sphere crosshead welded to a tapered shaft and applied to the specimens at a constant speed of 2 mm/min and at an angle of 30° to the long axis of the tooth. Maximum fracture loads were recorded in Newton with statistical purposes. Fractured specimens were assessed for failure modes: Catastrophic fractures (non-reparable, below the CEJ) and non-catastrophic fractures (reparable, above the CEJ). Classification was based on an agreement between three examiners.

### 2.7. Tridimensional Marginal Gap Analysis

**Figure 2:**
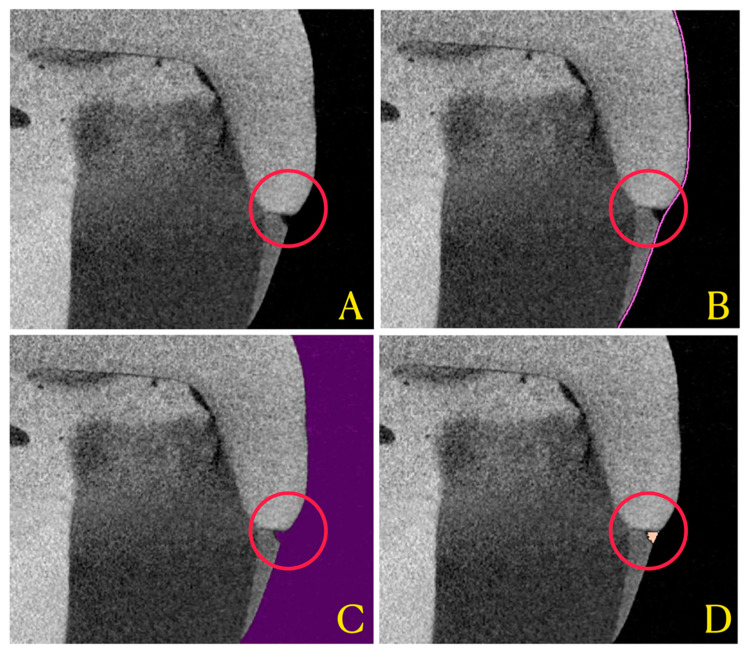
Applied workflow for 3D interfacial gap analysis. (**A**) represents the obtained micro-CT reconstructed image, imported in the segmentation software (Mimics 23, Materialise, Belgium). (**B**) shows the region of interest (ROI) defined by the software for gap analysis (pink line). (**C**) shows the void thresholding performed (violet mask) that defines “void” concept through all samples. (**D**) shows the intersection between the ROI and the void mask, ultimately representing interfacial gap (orange mask).

To reveal marginal gap progression between the indirect restoration and the tooth after cyclic loading, a tridimensional method of analysis was used. Through a dedicated software (Mimics Medical, ver. 23.0; Materialise, Belgium), thresholding of voids surrounding the restoration was performed automatically to include marginal voids only. Volumetric calculation of the resulting mask was performed by the software, and overall volume data of the residual marginal gap, expressed in mm^3^, were collected (Figure 2).

### 2.8. Statistical Analysis

In order to examine the effects of the study factors (core build-up and CAD/CAM materials) and the interactions between them on the marginal gap progression and the fracture resistance, a two-way analysis of variance test (ANOVA) was conducted. Post-hoc pairwise comparison was performed using Tukey test. All statistical analyses were performed using a software (STATA 12, ver. 12.0; StataCorp, College Station, TX, USA) and differences were considered significant for *p <* 0.05.

## 3. Results

### 3.1. Marginal Gap Variation

Results of marginal gap variation, expressed in mm^3^ (T1 after cyclical intermittent loading minus baseline T0) are reported in Table 2. Results of the ANOVA test showed that marginal gap was significantly influenced by the core build-up (*p <* 0.001) but not by the restorative material employed (*p =* 0.59). The interaction between the factors showed a significant influence on the marginal gap variation (*p =* 0.039). Tukey post hoc revealed that the fiber post presence promoted better gap results (lowered gap) compared to the resin composite core alone, apart from restorative material.

### 3.2. Fracture Resistance

Mean fracture resistance to static load, expressed in N, obtained in different groups was reported in Table 3. Two-way ANOVA test showed that fracture resistance was significantly related to the CAD/CAM material employed (*p <* 0.001) but not the FPSbu (*p =* 0.63). The interaction between the factors showed a not significant influence on the fracture resistance (*p =* 0.84). Tukey post hoc revealed that CS and CD groups had no statistical difference to each other and higher resistance than GB. Registered fracture patterns are reported in Table 4.

### 3.3. SEM Qualitative Analysis

**Figure 3:**
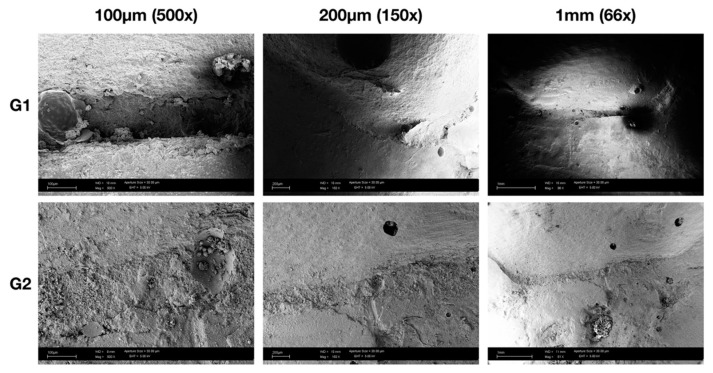
Representative SEM micrographs of the mesial surface from two random samples at different magnification. It’s possible to notice that both present marginal degradation after cyclical intermittent loading, mainly at the corners of the box area.

**Figure 4:**
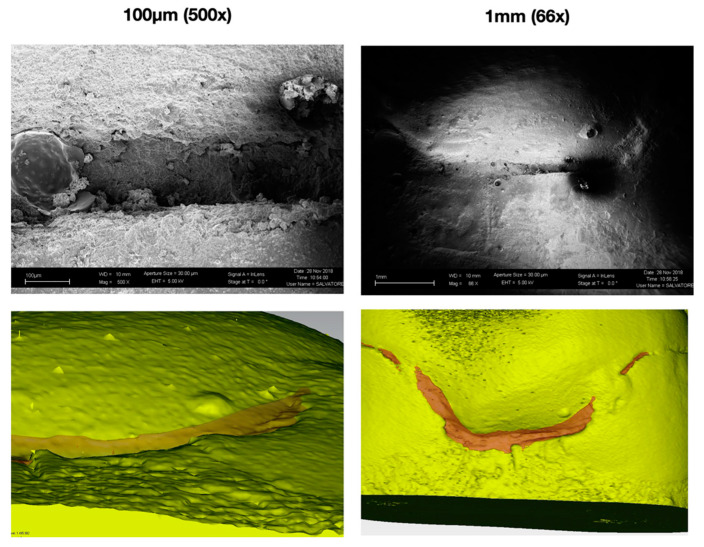
Previous sample from Figure 3 aside of micro-CT tridimensional gap analysis. For the present figure, images have been imported to an external software (Geomagic Qualify 12, 3D Systems, Rock Hills, SC, USA) and the analysis limited to the single box area for better visualization. Yellow volume represents the tooth-restoration complex, while the transparent red volume represents the marginal 3D gap that was calculated and analyzed.

SEM micrographs of adhesive margins showed marginal gaps after mechanical loading in all groups (Figure 3). Independently of the buildup, with or without fiber post, and the indirect restorative material tested, the typical localization of gaps was mainly located in mesial and distal areas of the interproximal box. It can also be noted that there is correspondence with the 3D reconstructions obtained from the renderings of the acquisitions via micro-CT (Figure 4), which, however, allowed for a quantitative and not only qualitative analysis of the marginal gap formation.

## 4. Discussion

Modern restorative procedures on ETT aim to improve their mechanical properties, which are inferior to those of their vital counterparts, while being minimally invasive to healthy dental tissues. To accomplish these goals, ETT are more and more frequently restored with adhesive approaches and partial luted restorations which represent a valid alternative to conventional crowns [11,37].

Based on the present study results, the first null hypothesis was partially rejected since FPSbu significantly influenced external marginal gap variation but not the residual fracture resistance after cyclic fatigue. A marginal gap opening was observed after intermittent loading in all specimens, corroborating in vivo and in vitro previous findings that showed how functional and parafunctional stresses, especially transversal forces, can cause degradation of the adhesive interface and a marginal gap variation [38,39]. Present findings showed how fiber post insertion within the composite build-up significantly reduced the gap opening. The higher flexural strength of fiber posts might mediate loads between dentin and CAD/CAM luted restoration, therefore resulting in a more homogenous stress distribution compared to composite-only build-up [40,41]. Moreover, in this study the indirect restorations performed were supported in all specimens by a composite build-up which had, independently of the fiber post presence, lower flexural strength and mechanical properties compared to the CAD/CAM material employed for the overlay fabrication. Thus, the build-up could represent the weakest part of the restoration complex together with the adhesive system. It is therefore reasonably to assume that a more rigid core build-up could bring mechanical benefits to the whole indirect adhesive restoration complex, reducing marginal stresses accumulation which could cause an opening during function. It should also be considered that the present study was designed to simulate both compressive and lateral forces during chewing simulation. Horizontal chewing patterns could produce a shear effect at the adhesive interface, with a high probability of causing progressive gap opening and debonding. The presence of a fiber post within the build-up might mitigate these forces, dissipating them among a wider adhesive interface and through the root canal system [42].

A positive interaction in terms of external gap opening was highlighted when zirconia reinforced lithium silicate was used in combination with the fiber post-supported build-up. It has been shown that resin-based composites blocks have inferior flexural modulus and flexural strength values compared to glass-reinforced ceramics [43,44]. According to that, CAD/CAM materials with higher flexural strength could better benefit from the augmented strength of the core build-up, which makes mechanical properties of the system more homogeneous, also considering the increased fragility of an ETT. Moreover, rigid materials could be more prone to transmit forces directly to the underneath structure [38], thus the ability of the fiber post to dissipate and distribute functional loads along the adhesive interfaces could be more evident.

SEM micrographs, above all at higher magnification, still offer a gold standard qualitative analysis of the external margins of adhesive restorations, as shown in Figure 4. Micro-CT, on the other hand, has the advantage of being a non-destructive method of analysis [45,46] that can offer not only a bi-dimensional, but also a tridimensional analysis of the sample before and after chewing simulation, therefore measuring gap progression in qualitative and quantitative ways. By contrast, SEM could be used to assess the presence of internal cracks even if sample sectioning is needed [47] and, when epoxy replicas are performed, only external margins can be inspected. Moreover, it must be noticed that micro-CT is also able to measure gap among the whole adhesive interface and not just external margins: Since forces also concentrate on internal edges, the study of internal gaps might be useful in the future. Analyzing gap localization through qualitative micrographs from SEM, it was also reported that margin opening seems to occur mostly in the interproximal boxes area. This can be considered in accordance with another study by Ausiello et al., which reported a high concentration of stresses in this area when applying forces on a finite element analysis (FEA) model [48].

After cyclic fatigue test, specimens were submitted to static fracture resistance test. Based on the study results, the second null hypothesis was partially rejected since the tested CAD/CAM materials significantly affected fracture resistance with GB showing a significantly lower resistance than other tested materials. This is probably related to the composition of the nano-hybrid CAD/CAM block, which has lower resistance compared to hybrid ceramics or zirconia-reinforced lithium silicate [49]. In general CAD/CAM composites, thanks to their more compact and cured tridimensional structure, show greater flexural and compressive strength values compared to traditionally layered composites. However, they are not still able to have a biomechanical behavior comparable to glass-reinforced ceramics [50]. Previous studies showed slightly different fracture resistance values [51], probably due to different build-up techniques and the different tooth preparation for the restoration. Furthermore, another reason for these inconsistencies with the present study results could related to the 30° angle applied during the fracture resistance test with the universal machine, which could affect the fracture values as well as the fracture pattern [52].

On the other hand, this in vitro study did not highlight a significant correlation between the fiber post-supported build-up and the residual fracture resistance, though the second null hypothesis was partially rejected. This is in accordance with a similar study conducted on endodontically treated premolars restored with partial ceramic restorations, which reported that the fracture resistance was not improved by the insertion of glass or quartz fibers posts [53]. Similar results were reported by Scotti et al., assessing composite onlay [54], and by Krejci et al. on several indirect adhesive composite configurations [55]. Moreover, a recent study by Magne et al. on all-ceramic leucite-reinforced glass ceramic crowns confirmed that insertion of a fiber-reinforced post does not enhance the load-bearing capacity of the tooth [56]. Thus, in terms of fracture resistance, the fiber post use could be considered clinically irrelevant compared to other factors such as the ferrule effect and the cuspal coverage itself.

For what concerns fracture pattern, a reduction of catastrophic failures in association with fiber post occurred in all groups. This is in accordance with a literature review by Goracci et al., which reported reduced risk of vertical root fractures when glass fiber post is applied [42]. Moreover, Newman et al. suggested that fiber posts might dissipate forces along the root canal, reducing stresses on the root and therefore preventing catastrophic failures [57]. It has also been hypothesized that, when forces exceed tolerance of the system, fiber posts might be able to concentrate stresses in the coronal portion, ultimately resulting in a repairable failure pattern [22,32]. This is also in accordance with a recent in-vivo review, which concluded that failures of fiber posts were mainly due to post loss of retention, compared to metal post that presented a higher amount of root fractures [58].

A limitation of the present study was the absence of thermal stresses during the cyclic fatigue test that could mimic intra-oral temperature changes: since composites and adhesives have a higher thermal contraction/expansion coefficient than hard tooth tissues, this might influence gap formation and progression.

## 5. Conclusions

Based on the results of the present study, it seems that the use of a fiber post within the composite build-up which support adhesive overlays in ETT had a significant positive effect for the external marginal gap opening after cyclic intermitted loading. Thus, from a clinical point of view, it could be speculated that its use could promote a marginal leakage reduction during oral function. On the other hand, different CAD/CAM restorative materials were not able to significantly affect the interfacial gap behavior. Another important finding was that a rigid restorative material, such as zirconia-reinforced lithium disilicate, seem to benefit the most from the insertion of a fiber post in terms of gap reduction. It is also important to consider how the tridimensional method used in this study to quantify the interfacial gap progression seems to give encouraging results.

Considering the residual fracture resistance after cyclic fatigue, Cerasmart and Celtra Duo better performed if compared to Grandio Blocks. However, the fiber post insertion was not a parameter which influenced the tooth-restoration complex resistance. Moreover, confirming previous studies, encouraging results on fracture pattern were found when fiber post was applied.

Further studies are necessary to confirm the obtained results, in order to offer precise protocols to clinicians regarding indirect partial adhesive restorations on ETT.

## Figures and Tables

**Table 1:** General description of the main materials used in the present study.

Material	General Description	Manufacturer	Composition
Grandioso X-Tra	Nanohybrid bulk resin composite	Voco	86% *w*/*w* filler content, Bis-GMA, UDMA, TEGDMAc
Cerasmart 270	Hybrid ceramic	GC	71 wt% silica and barium nano glass, Bis-MEPP, UDMA, dimethacrylate co-monomers
Celtra DUO	Zirconia reinforced lithium disilicate	Dentsply	58% silicon dioxide, 10.1% crystallized zirconium dioxide, 10% zirconium dioxide, 5% phosphorous pentoxide, 2.0% ceria, 1.9% alumina, 1% terbium oxide
Grandio Blocks	Nanohybrid reinforced composite	Voco	86% *w*/*w* inorganic filler in a polymeric matrix
Rebilda Post #15	Glass fiber reinforced post	Voco	Solid composite of glass fibers, inorganic fillers, PDMA

**Table 2:** Mean interfacial gap variations ± standard deviation, expressed as mm^3^, for each subgroup. Same superscript letters indicate no significant differences.

	CS		GB		CD	
	Fiber Post (−)	Fiber Post (+)	Fiber Post (−)	Fiber Post (+)	Fiber Post (−)	Fiber Post (+)
Marginal Gap Variation (mm^3^)	0.52 ^a^ ±0.08	0.44 ^b^ ±0.07	0.52 ^a^ ±0.06	0.45 ^b^ ±0.04	0.59 ^a^ ±0.09	0.41 ^b^ ±0.05

**Table 3:** Mean fracture resistance ± standard deviation, expressed as Newton, for each subgroup. Same superscript letters indicate no significant differences.

	CS		GB		CD	
	Fiber Post (−)	Fiber Post (+)	Fiber Post (−)	Fiber Post (+)	Fiber Post (−)	Fiber Post (+)
Fracture Resistance (N)	1481.21 ^a^ ±195.27	1576.22 ^a^ ±220.51	1136.43 ^b^ ±202.37	1203.86 ^b^ ±149.88	1351.52 ^a^ ±208.08	1484.45 ^a^ ±179.05

**Table 4:** Fracture patterns for each subgroup.

		CS		GB		CD	
		Fiber Post (−)	Fiber Post (+)	Fiber Post (−)	Fiber Post (+)	Fiber Post (−)	Fiber Post (+)
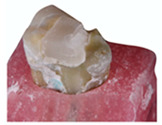	Catastrophic	5	4	4	2	3	1
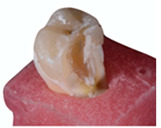	Non-catastrophic	3	4	4	6	5	7
